# An approach to making life sciences FAIR—FAIR-DS as a tool for *Aspergillus fumigatus*

**DOI:** 10.1093/database/baaf082

**Published:** 2026-01-02

**Authors:** Sibbe Bakker, Mariana Santos-Silva, Johanna Rhodes, Sijmen Schoustra, Bas Zwaan, Anna Fensel

**Affiliations:** Laboratory of Genetics, Wageningen University & Research, Droevendaalsesteeg 1 Building 107 6708 PB Wageningen, the Netherlands; Wageningen Data Competence Center (WDCC), Wageningen University & Research, Droevendaalsesteeg 4, 6708 PB Wageningen, the Netherlands; Laboratory of Genetics, Wageningen University & Research, Droevendaalsesteeg 1 Building 107 6708 PB Wageningen, the Netherlands; Department of Medical Microbiology, Radboudumc, Geert Grooteplein Zuid 10 6525 GA Nijmegen, Nijmegen, the Netherlands; Laboratory of Genetics, Wageningen University & Research, Droevendaalsesteeg 1 Building 107 6708 PB Wageningen, the Netherlands; Laboratory of Genetics, Wageningen University & Research, Droevendaalsesteeg 1 Building 107 6708 PB Wageningen, the Netherlands; Wageningen Data Competence Center (WDCC), Wageningen University & Research, Droevendaalsesteeg 4, 6708 PB Wageningen, the Netherlands; Artificial Intelligence Chair Group, Wageningen University & Research, Droevendaalsesteeg 1 Building 107 6708 PB Wageningen, the Netherlands

## Abstract

Adhering to Findable, Accessible, Interoperable, and Reusable (FAIR) principles ensures that data is shared in ways that maximize reusability. However, not all researchers in biological fields, particularly those that rely on manual data entry, have embraced these principles, particularly when it comes to adhering to relevant ontologies. This hampers data sharing due to missing metadata, limiting reuse. This study addresses the production of FAIR data for mycology, a field characterized by high manual data entry demands. For this field, we propose specific data standards to enhance reusability in the *Aspergillus fumigatus* field and beyond, emphasizing their importance for fostering collaboration and accelerating scientific progress. We adopted the FAIR-Data Station (FAIR-DS) solution to this new domain and demonstrated how to improve the FAIRification of new types of complex life science datasets. For individual researchers, FAIR-DS simplifies data uploads to repositories while ensuring critical metadata is preserved. Following our approach, we have converted two types of datasets—spore counts and a compost monitoring programme on Dutch farms—into FAIR ontology-compliant formats and published them as open data.

## Introduction

Effective data management is a fundamental aspect of modern life sciences. Digital systems have largely replaced traditional paper-based data storage. Consequently, researchers are now expected to manage and analyse large datasets while ensuring compliance with data sharing mandates from funding bodies and academic journals [1]. Hence, the increasing volume and complexity of data sets introduce new challenges. A significant majority of researchers (85%) acknowledge the importance of data sharing [2], but many struggle to convert their data into standardized and reusable formats. Furthermore, 29.8% of the researchers still store their data in unsecured local environments [3], posing risks to data integrity and accessibility.

Zenodo or the European Nucleotide Archive (ENA), and the National Center for Biotechnology Information (NCBI) are examples of community efforts aimed at tackling some of the previous needs by contributing to the establishment and usage of common data repositories. However, recent developments in data-sharing platforms were not sufficient to ensure data maintenance in standardized formats. Hence, finding research data sets in FAIR formats remains a major challenge. This issue is exemplified by metadata inconsistencies in BioSamples stored within NCBI and ENA databases [4], where more than 89% of metadata entries contain non-standardized values and only 27% of Boolean values are correctly formatted. The consequences of poor data standardization are evident in epidemiological research, as seen during the COVID-19 pandemic [5], where missing metadata, such as collection dates and geolocation, hindered data integration and analysis.

To demonstrate how these standardization and data management challenges can be addressed in a field with complex data handling, we tested the application of a data analysis tool to transform complex data sets of *Aspergillus fumigatus* into FAIR formats.

### *Aspergillus fumigatus* as use case for data management strategies

Maintaining data in standardized formats with comprehensive metadata is an increasingly pressing challenge in fungal pathogen research. As a priority organism for the World Health Organization [6], *A. fumigatus* is a relevant use case for investigating metadata standards for mycology. Due to its importance as a human pathogen, *A. fumigatus* is an opportunistic ascomycete pathogen with remarkable adaptability, thriving as a saprophyte in organic waste and infecting plants as well as immunocompromised animal hosts [3, 7]. *Aspergillus fumigatus* is then a saprophite able to connect agriculture and medicine, policy and industry. Stakeholders from various sectors are part of the azole ecosystem, and therefore, need to collaborate. Currently, *A. fumigatus* is developing resistance towards azole antifungals, which poses a serious threat to public health. With the increasing use of immunosuppressive therapies, particularly in organ transplantation and cancer treatment, the number of immunosuppressed individuals is increasing [7].

This bridge across sectors, involving diverse stakeholders, is also translated into the complexity of data sets. A rapid increase in resistance to azole fungicides is not being followed by a fast development of new azole drugs. Therefore, it is vital to find data management tools to achieve an effective understanding of *A. fumigatus* and control its resistance to azole fungicides in both agricultural and clinical settings.

To investigate the environmental origins of resistance to azole in *A. fumigatus*, numerous studies have attempted to correlate resistance patterns with environmental factors [8–10]. However, the lack of standardized data practices limits the efforts of integration of datasets from different sources. Typically, researchers deposit sequence data in BioSample [11] while storing metadata in supplementary Excel files, leading to fragmentation and hindering epidemiological analyses. Addressing these challenges requires adherence to the Findable, Accessible, Interoperable, and Reusable (FAIR) principles outlined by Wilkinson *et al*. [12]. Several metadata management tools, including COPO [13], CEDAR [14], investigation, study, and assay (ISA)-Tab [15], and the FAIR Data Station (FAIR-DS) [16], have been developed to support the creation of FAIR-compliant datasets. The FAIR-DS differs in that it uses Excel, which is commonplace in the data entry workflow of a biologist.

In this study, we demonstrate how to interlink existing research data on *A. fumigatus* to facilitate re-analysis and improve data interoperability. Since immunosuppressive therapies, particularly used during organ transplant or cancer treatment, are more effective, the number of immunocompromised people is increasing [7]. More frequently, *A. fumigatus* is gaining resistance to antifungal drugs due to environmental exposure to agricultural fungicides or prolonged clinical exposure through antifungal therapy. This results in significant health risks to the immunocompromised population. Resistance primarily occurs via mutations in *cyp51A*, the target of azole antifungal drugs; the most common mutation, TR_34_/L98H, has been described globally [17, 18].

To identify the source of resistance to azoles in *A. fumigatus*, various efforts have been made to relate this to environmental factors [10, 19, 20]. Generally, researchers upload their sequence data to BioSample and upload their metadata as an Excel file, which is part of the supplement to their paper. The lack of data standardization makes it difficult to interoperate with datasets from different environmental sources, adding complexity to discovering and analysing the epidemiology of *A. fumigatus* azole resistance from published datasets.

### Transforming the FAIR-DS into FAIR *A. fumigatus* data

**Figure 1.**
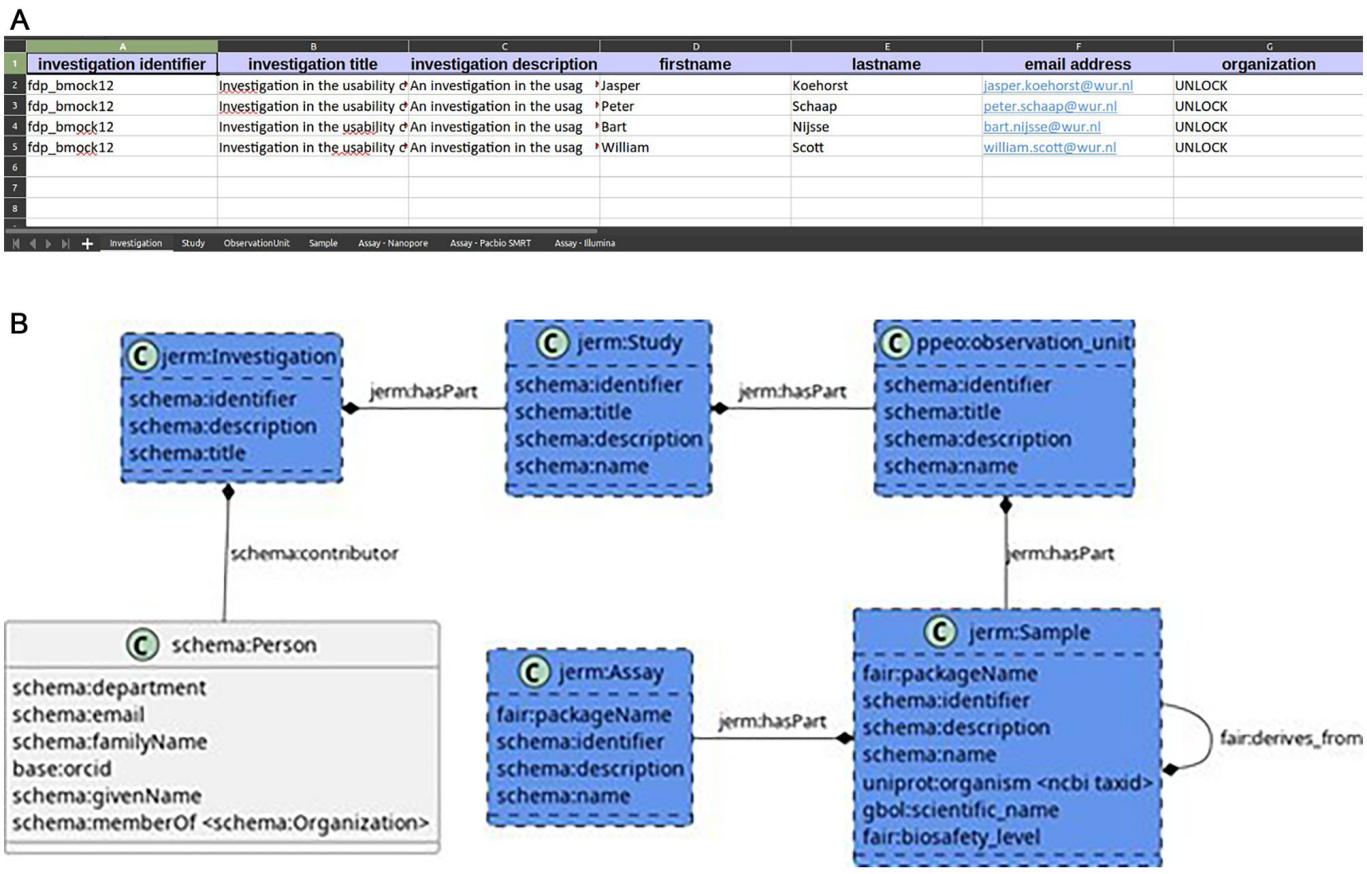
Overview of the FAIR-DS. The FAIR-DS models five classes (B), the investigation, study, observational unit, sample, and the assay. Each class is given a sheet in the Excel document (A). In these sheets, the fields of the class can be filled in. Each instance of the class has a row in the Excel sheet.

In this paper, we show how to interlink existing research data of *A. fumigatus* to facilitate re-analysis. We describe the introduction of FAIR data tools in a new domain using the FAIR-DS, a lightweight metadata management tool developed for system biologists by Nijsse, Schaap, and Koehorst [16]. The FAIR-DS uses a modified version of the ISA format [21] to allow the entry of laboratory data as Excel files and the conversion to resource description framework (RDF) files (Fig. 1A). The FAIR-DS tool builds on the ISA framework to clarify the terms ‘sample material’ and ‘source material’ that Nijsse, Schaap, and Koehorst [16] found confusing to users of FAIR-DS. The ‘observational unit’ from plant phenotyping experiment ontology is used to replace the ‘source material’, and the ‘sample material’ is replaced by the ‘sample’ from just enough results model (Fig. 1B). The minimal metadata models for each of these five classes, which are used to verify the data converted by FAIR-DS, are defined as Excel sheets. In these Excel sheets, it is possible to define the data types and valid patterns of input data. The minimal metadata model is used to reject or accept a dataset specified by the researchers.

Like the BioSample database, the FAIR-DS uses metadata packages for each of the five mentioned classes. A metadata package is a Table with a set of controlled columns (the metadata terms). The FAIR-DS verifies whether all the required terms of a package are present and fit a given pattern. For example, for a soil sample, the sampling depth must be indicated before the input metadata can pass the FAIR-DS validation. In the FAIR-DS, the validation patterns are defined as a set of Tables: a ‘terms’ Table, where the name of each term, together with its pattern and documentation, is described. Additionally, a link to an ontology can be given. The patterns are defined in a ‘regex’ Table. The packages are defined for the five FAIR-DS classes (Fig. 1B).

Since the basic structure of investigation, study, observational unit, sample, and assay used by the FAIR-DS is common to many fields, the FAIR-DS is hypothesized to be useful outside of the system biology domain. Due to its simplicity for the user and Excel-based data entry system, the FAIR-DS is a useful test case to introduce FAIR data tooling into a domain where FAIR standards have not yet taken place. In this article, we also demonstrate the process of guiding researchers in the integration of FAIR data management tools from the beginning of their research project. By using two datasets made FAIR post hoc, we show the directions to where metadata standardization in the *A. fumigatus* domain is most needed, such as location data, experimental design, among others.

## Methods

### Introducing the FAIR-DS to the *A. fumigatus* domain

A set of basic requirements regarding the FAIR data management workflow was discussed and designed during meetings with other researchers in the field of *A. fumigatus*, bioinformatics, and data management. This initial work on the FAIR-DS was presented at the annual Azole Resistance Meeting (Wageningen, November 2023) [22]). At this international meeting, the applications of the FAIR-DS as a tool to the *A. fumigatus* domain were demonstrated, and it was shown how the FAIR-DS related to the requirements. Following this annual meeting, the FAIR-DS was introduced to a group of five mycologists to test how usable it is for data management outside the field of systems biology. The researchers were introduced to the software with presentations and one-on-one sessions (four sessions per person, each an hour long) where they were given guidance on how to use the FAIR-DS in their research. To determine the needs of each use case, interviews were conducted: each user was asked to provide an outline of their research topic, questions, and methods. Protocols and raw datasets were also investigated. During the interview, the standardization problems with the user and their current data management practice were discussed.

Using the schema based on the FAIR-DS ontology, the entire *A. fumigatus* study was merged into a single dataset. It is available at https://doi.org/10.5281/zenodo.14021468.

A new standard of data entry was created with the user. First, a draft was made, which was shown to the user for feedback. Based on the feedback, the draft was changed. These rounds continued until there was a minimally useful standard. The standards were represented as unified modeling language (UML) (https://sparxsystems.com/resources/tutorials/uml/datamodel.html) diagrammes using PlantUML [23]. Two methods were used to write RDF in the FAIR-DS format: for the SchimmelRadar data sets by Kortenbosch *et al*. [8], FAIR-DS Excel sheets were used as an intermediate format, which was uploaded to a local instance of the FAIR-DS validation and conversion web service. The more complex Monitoring dataset by Mariana Santos Couto Silva and Sibbe Bakker [24] was made to fit the FAIR-DS format by writing triples with the tarql programme.

### SchimmelRadar data

A local installation of the FAIR-DS (v1.0.212) was used to validate Excel sheets and host minimal metadata models. (As is shown in the repository: https://git.wur.nl/standards/aspergillus-data-standardisation/fairds-local-instance.) The R programming language [25] was used with the tidyverse [26] and openxlsx [27] to convert the dataset published by Kortenbosch *et al*. [8] to the FAIR-DS Excel format.

### Monitoring data

The compost data was collected in 2021 [24] in various Excel formats at the request of P. Adema [19]. To make the data adhere to FAIR standards, each dataset was examined and cleaned so that inconsistencies in the formatting and coding could be fixed.

Using Snakemake [28], a pipeline was made to make the monitoring data fit the FAIR-DS format. This method was chosen instead of the FAIR-DS web service in order to have more control of the schema and to be able to easily re-run the pipeline. Using the readxl [29] and the tidyverse [26] in R [25], each Excel workbook was exported to a CSV format. Then, using tarql [30], the Tables were transformed into RDF according to an extended FAIR-DS ontology. Using Oxigraph [31], the RDF was queried.

## Results

### Using the FAIR-DS for the management of *A. fumigatus* laboratory data

Interviews were conducted with researchers working with *A. fumigatus* to define initial requirements for data set management. Follow-up discussions with researchers and other stakeholders were scheduled during weekly team meetings within the Genetics department and annual international meetings (such as the https://www.nwo.nl/projecten/taauc51336), for an effective co-creation method. During these discussions, the requirements were set out as follows: (1) the workflow must be compatible with the Excel-based workflow that is already used, and (2) the workflow must ensure that ownership of the data remains with the user. Meetings with stakeholders from various sectors and diverse roles (academia, industry, consultants, farmers) were organized to validate these requirements.

At the annual Azole Resistance Meeting of 2023 (Wageningen, November 2023), the FAIR-DS was introduced to a group of mycologists to test its usability for data management outside the system biology field. The researchers were introduced to the software with presentations and one-on-one sessions where they were given guidance on using the FAIR-DS in their research. During these sessions, the researchers were asked to prepare an example dataset for which FAIR-DS templates were designed with them. One notable use case was the templating data entry schemes for the Kortenbosch *et al*. [8] air sampling method developed for the Dutch *SchimmelRadar* project [32]. Another use case was the data standardization of *A. fumigatus* monitoring data from various Dutch farmers [24].

**Figure 2.**
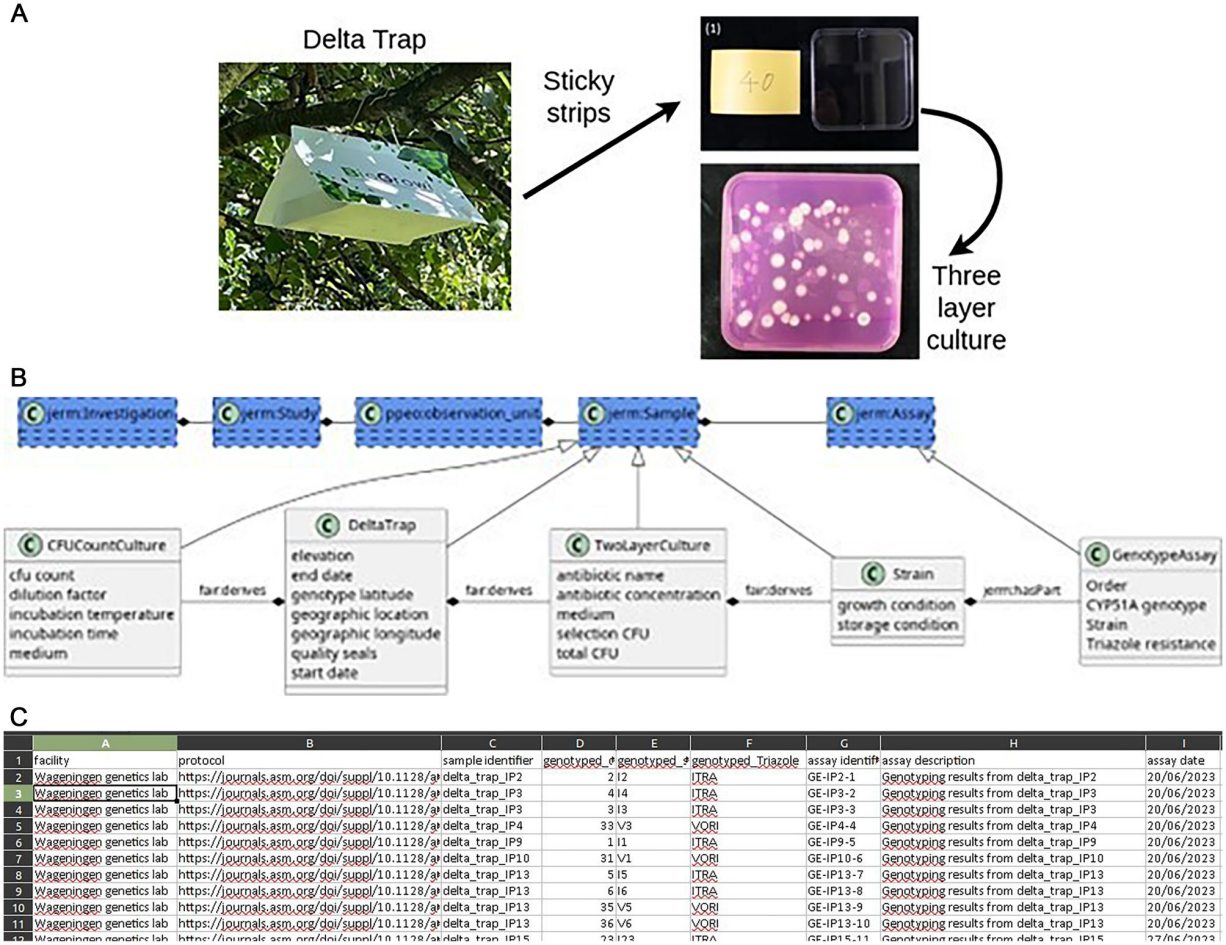
Overview of the SchimmelRadar method. (A) A DeltaTrap with three sticky strips is used to capture spores from *A. fumigatus*. These strips are incubated in selective conditions to obtain CFUs of *A. fumigatus*. The schema of the SchimmelRadar project. (B) Four subclasses of sample are made: CFUCountCulture, DeltaTrap, TwoLayerCulture, and Strain. One assay, the GenotypeAssay is added. Only the relevant fields that are added to the default classes are shown. (C) Example of the dataset.

Recently, a method to reliably sample the resistance fraction of *A. fumigatus* spores present in the air (Fig. 2B) was developed by Kortenbosch *et al*. [8]. This method samples fungal spores using three sticky strips repurposed from 96-well plate seals over four weeks. Two strips are used to determine the fraction of the total spores to two antifungals: itraconazole and voriconazole.

This is achieved by incubating the strip into a minimal medium that selects for *A. fumigatus*. After the *A. fumigatus* spores have germinated, the ‘strip total CFU’ is determined. Then a medium with an antifungal (either voriconazole or itraconazole) is added, and the spores that grow here are ‘resistant’. Using the ‘total’ and ‘resistant’ spore count, a resistance fraction will be determined. For a better estimate of the total amount of spores that landed on the DeltaTrap, the last of the 3 strips is used for a colony forming units (CFU) count culture on medium without any antifungals. Lastly, a polymerase chain reaction (PCR) assay is used to discern the *cyp51A* genotype of the *A. fumigatus* isolate.

### Our solution—FAIR-DS 2.0

To improve data management for this experiment, we created 4 additional packages for the FAIR-DS that describe the various experiments of the SchimmelRadar method. All of these FAIR-DS packages are based on the default package, which captures the minimal information needed for quality assessment and data analysis (Fig. 2B). The SchimmelRadar methodology has three sample types: the DeltaTrap, which holds three sticky strips, from which the two TwoLayer cultures and a CFU count culture are started. A PCR genotyping is made on one of the cultures derived from the DeltaTrap. Kortenbosch *et al*. [8] use geographical region as the observation unit, this is entered in the FAIR-DS Excel sheet as a set of region names and identifiers. For each observation unit, the DeltaTrap samples are entered with the required geolocation data. Other samples, such as the TwoLayer culture and the CFU count culture, are added that link to the observational unit, but with the added information that they derive from a specific DeltaTrap.

The DeltaTrap package is the core of the SchimmelRadar metadata packages. It tracks the start and end dates of the sampling period; the location, including the name of the location and coordinates; and the height of the DeltaTrap. Optionally, the quality of the sample can be recorded, such as contamination or if the packaging is broken. The microbial taxa of interest are specified as an NCBI taxid: 746 128 (*A. fumigatus*) should be entered. From this DeltaTrap package, TwoLayer cultures, CFU count cultures, and genotype assays are derived. In the TwoLayer culture package, parameters for the TwoLayer culture incubation are recorded by linking to a protocol document. Minor variations on the protocol, such as antibiotic type and concentration, are also mandatory to include. The antibiotic name is entered as a label from the ARO ontology [33]. The CFU count for this culture must be given before and after adding the selective medium. The CFU count culture serves as a control and is used to determine how many colonies of *A. fumigatus* are isolated in total. It documents which medium was used and the number of CFU observed on the plate.

The PCR based genotyping assay used by Kortenbosch *et al*. [8] is used to assess mutations related to azole resistance of an *A. fumigatus* strain from a specific plate. This assay is documented by using the default FAIR-DS template for an assay and referring to the protocol released by Kortenbosch *et al*. [8]. Additionally, the FAIR-DS terms for forward and reverse primers are used to document the PCR.

New SchimmelRadar data is entered into the FAIR-DS format by starting a FAIR-DS instance with the SchimmelRadar packages (see the ‘Availability statements’ section). Then the required packages can be selected in the metadata configurator’ menu. The Excel sheet generated with the FAIR-DS Fig. 2C may be filled in and submitted to the FAIR-DS.

**Figure 3.**
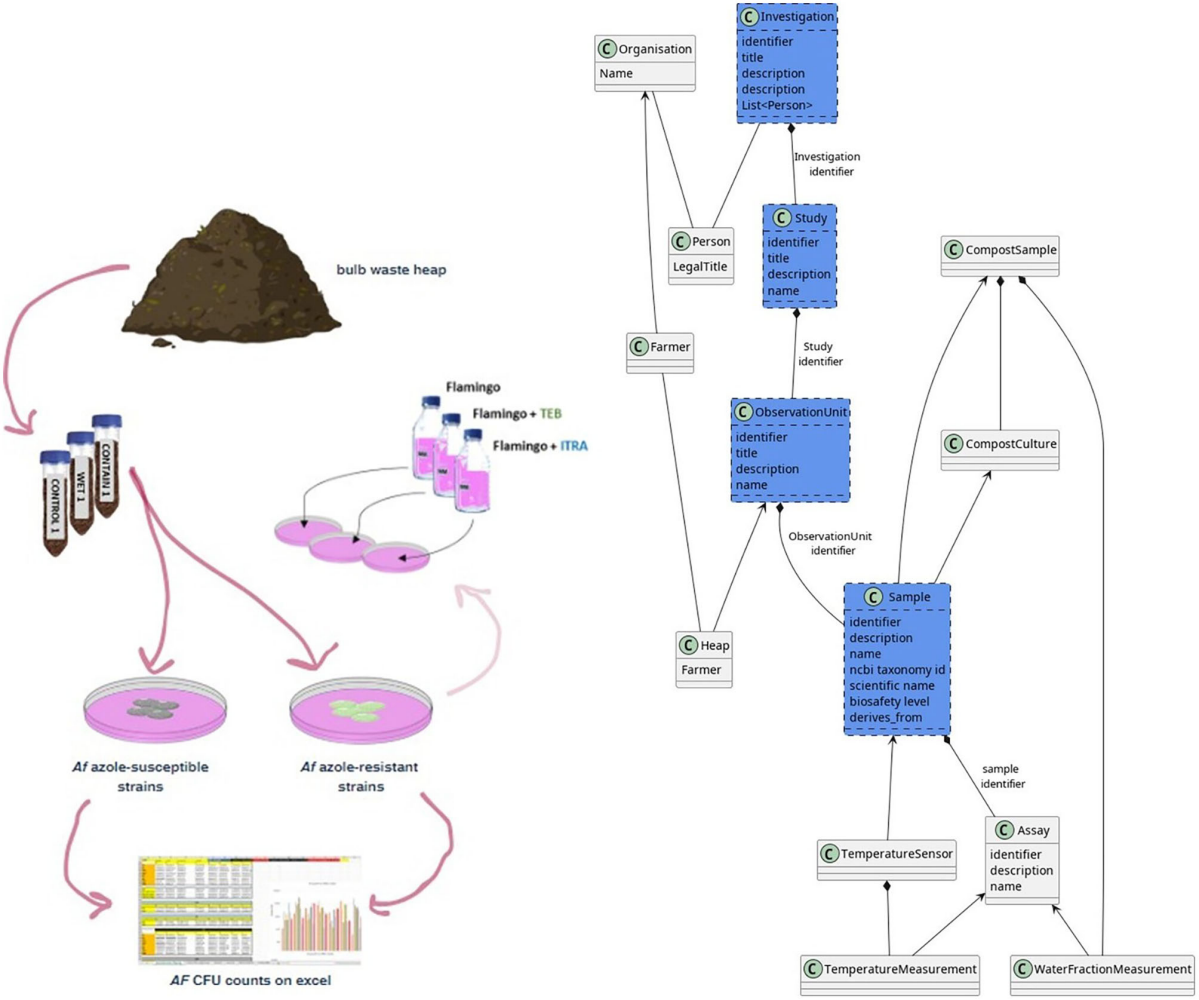
Methodology and schema for the Monitoring dataset. Steps used for analysing azole resistance in bulb-based compost in the laboratory (describe don the left side), and the methodology for data analysis (described on the right).

To show that the FAIR-DS approach can generalize to different datasets, we also applied it to a dataset of Compost heap samples collected by Mariana Santos Couto Silva and Sibbe Bakker [24] in the *A. fumigatus* Monitor Programme. This experiment aimed to establish what the relationship was between the watering regime and *A. fumigatus* growth. This was achieved by conducting a survey on colony-forming units in four farms in Noord-Holland, where compost samples were taken and cultivated on Flamingo medium [34] (Fig. 3B).

## Discussion

### Suitability of the FAIR-DS

The FAIR-DS is a tool for managing and validating research data with Excel sheets and was designed to target wet-lab scientists [16]. Unlike other systems that typically focus on either metadata schema design or data repository implementation, the FAIR-DS distinguishes itself by covering the full workflow—from experimental planning (selecting metadata packages and generating data entry sheets) to data capture and archiving in a queryable database (Table 1). By comparing strengths and limitations of existing data collection and sharing methods in relation to FAIRness and usability, it is clear why traditional tools such as Excel and Electronic Lab Notebooks (ELNs) remain popular. Both methods offer accessibility and familiarity to the user, however lack the capacity for metadata standardization, long-term preservation, and FAIR data structuring. Conversely, domain-specific infrastructures such as COPO, CEDAR, and ISA-Tab have made significant advances towards FAIR compliance. The comparison in Table 1 also reflects that, despite the positive aspects of working with these tools, there are challenges still to overcome, such as often requiring specialized expertise and time for adaptation between disciplines. While CEDAR promotes standardized metadata creation and can interface with external repositories, it does not provide in-platform long-term data archiving. Platforms such as GitHub and GitLab surpass in version control and collaborative functionality but are not primarily designed for research data management [35]. Both these platforms, together with Zenodo, are archiving tools for small data sets, not FAIR data tools. GeneBank was also excluded from this comparison since it is not a FAIR data tool but a domain-specific repository (hence, an archive tool as well) specialized in genomic data. In contrast, the FAIR-DS approach was conceived to integrate workflow coverage, FAIR-compliant metadata generation, and long-term data stewardship while maintaining accessibility for users with varying technical backgrounds. This highlights the potential of FAIR-oriented tools to bridge the gap between usability and compliance within diverse research environments.

**Table 1.** Comparison of data collection methods (ELN, Excel, COPO, CEDAR, ISA-Tab, and our FAIR-DS-based solution—FAIR-DS 2.0) regarding FAIRness and usability.

Traits of each method	ELN	EXCEL	COPO [13]	CEDAR [14]	ISA-tab [36]	FAIR-DS [16] 2.0
Full workflow coverage	Partial	No	Yes	Partial	Partial	Yes
Enables cross-domain collaborations	Partial	Partial	Partial	Partial	Partial	Yes
Possibility to edit offline	Yes	Yes	No	No	Partial	Yes
Long-term archiving	Yes	No	Yes	No	Partial	Yes
FAIR (meta)data standardized formats	No	No	Partial	Yes	Yes	Yes
Protocols/guidelines available online	Yes	Yes	Partial	Partial	No	Yes
Operable as a standalone system	Yes	Yes	Partial	Partial	Yes	Yes
Data exportable in interoperable format	Yes	Yes	Partial	Partial	No	Yes

The term *Partial* denotes that the feature is only partially implemented or dependent on external services or manual intervention to achieve full FAIR compliance.

As a published program, the FAIR-DS has a stable interface. However, to improve its usability as a widely used data sharing and management tool, some aspects should still be improved. For instance, at the scheme level, the validation of patterns and the presence of important fields are not sufficient: no cardinality restrictions can be enforced on the links between packages, and so, it is not yet possible to check whether, for example, a DeltaTrap has TwoLayer culture samples and one CFU-count culture. Similarly, the current implementation does not validate whether user-entered terms are correct within the chosen ontology, limiting interoperability with external systems. Before the FAIR-DS can become more widely used, these improvements must be included in the ontology. Furthermore, the ontology terms of the FAIR-DS are still under stabilization, which means that the way datasets are interlinked may evolve over time.

Our solution FAIR-DS 2.0 represents a more reliable method of data collection and sharing than simply attaching Excel sheets to publications or circulating them informally among stake- holders, despite its current limitations. The direct benefit for laboratory researchers is that they receive a clear template for data entry, while colleagues can access and reuse data more efficiently. The RDF generated through FAIR-DS is particularly valuable for sharing and analysing data, especially since the underlying Excel sheets may appear complex to laboratory scientists. Some programming knowledge is still required to fully exploit FAIR-DS’s capabilities, yet with the advent of generative artificial intelligence, these skills are becoming more accessible to a wider audience. Taken together, the FAIR-DS offers a pragmatic and extensible entry point into FAIR data practices, while future improvements addressing schema, implementation, and archiving concerns will increase its impact across both the life sciences and the informatics communities.

### The human factor

More than ever, scientists have multiple resources to understand the need for collecting data in a FAIR format and to learn data management and coding skills. However, the adoption of data management tools requires the commitment of researchers, since a change in their working culture is necessary. Within the departments and research groups approached to test the FAIR-DS tool, no changes in FAIR practices were implemented. From our direct contact and open feedback from tests, meetings, and group discussions, it was clear that all researchers found metadata to be important; however, they lacked the time and expertise to implement good metadata practices. This aligns with previous findings: Jiao, Li, and Fang [37] found a steady increase in the use of data sharing repositories in life sciences. Hughes *et al*. [38] also recommend improving the FAIR data training for researchers. At the same time, it is important to recognize that metadata practices are not only a matter of lacking time or expertise but are often deprioritized in day-to-day research workflows. Ensuring FAIR data is therefore not solely the responsibility of funding agencies; researchers also hold responsibility for documenting and sharing metadata, much of which should already exist within their internal records. For a sustainable use of data in a rapidly developing and globalized technological world, it is vital to support more initiatives aimed at data reusability, while funding bodies should also require proper metadata documentation as a condition of support. Long-lasting and substantial changes in research culture take time but may not even be possible without national and international entities acknowledging their role in providing structural support and funding for a more FAIR data world.

### Recommendations for the *A. fumigatus* field

Last year, the Joint Programming Initiative on Antimicrobial Resistance (JPIAMR) awarded 2000 000 euros to a global *A. fumigatus* monitoring project [39]. Monitoring methods, such as those of Kortenbosch *et al*. [8], could be employed to detect *A. fumigatus* resistance on a wider scale. Such projects will be most successful if not only the data collection is standardized but also the analysis workflow. Before starting experimental work that may result in a dataset that will have long-term significance, we advise drawing a data management plan that considers data sharing and ontologies and the ontology-adherent FAIR data production to be implemented by design at the core of the data creation. In this manuscript, we have described the FAIR-DS ontology to provide a solid foundation for such implementation and research.

### Concluding remarks

Our team, inspired by the growing relevance of azole resistance in *A. fumigatus* and the need of promoting FAIRification in this filed, applied an existing programme to generate FAIR data in a new domain. This programme, the FAIR-DS, was originally developed for system biologists by Nijsse, Schaap, and Koehorst [16]. Considering the limited timespan of the project (one year), a complete widespread change in data management culture was not expected. Moreover, other data standardization initiatives have reported similar levels of progress; for example, Verbeke [40] built a FAIR and open database for water purification research, had not yet received active submissions a year after its release. In our case, differences in needs and working cultures between departments posed a challenge: while biologists in the systems biology department manage and analyse data with support from bioinformaticians, mycologists in the genetics department are often solely responsible for their own data management and analysis, making the adoption of specialized technologies such as RDF more difficult. Achieving lasting cultural change requires more time and the development of additional FAIR tools tailored to diverse user contexts. By sharing this work, we aim to encourage both broader adoption of FAIR practices and the continued exploration of FAIR-aligned tools, even when immediate uptake may be limited.

Regarding the common implementation of Excel as a data collection method and sharing method, since it is a pragmatic and accessible choice, this decision raises several considerations. A clear distinction should be made between schema and implementation, and both schema and functions should be conceived in ways that allow transformation into and/or compatibility with generic widespread web formats and languages (e.g. HTML, JavaScript, or PHP). Long-term archiving also requires attention: Excel is proprietary and unsuitable as a preservation format, and simple CSV storage is insufficient without detailed documentation of content and functions. Because proprietary formats are often regarded critically within the scientific community, their use requires careful documentation and sustainability planning. By analogy to microbial cultivation, where standard steps such as separating mixed cultures into pure cultures and cloning are considered fundamental, the schema and implementation of FAIR-DS should also reflect such basic requirements, even if they are not strictly necessary in every setup [41].

This case study highlights how domain-specific research practices and data cultures critically shape the adoption of FAIR tooling. The limited uptake of FAIR-DS in the *A. fumigatus* research community was not only due to technical barriers but also to differing organizational practices, expectations, and data management responsibilities between research fields. This suggests that FAIR implementation efforts need to go beyond technical solutions and consider the socio-technical context of research communities. Tools and standards may need to be adapted—not only in their design—but also in how they are embedded within existing workflows, incentive structures, and support systems. Future work could explore co-creation approaches with researchers to develop lightweight, user-friendly solutions aligned with the everyday practices of diverse scientific domains. This work shows the potential of adapting pre-existing tools to make science more FAIR, beyond azole resistance. However, only by focusing more on research behaviour and its needs as a user, we can have a positive impact towards a more FAIR science and a fairer world.

## Supplementary Material

baaf082_Supplemental_File

## Availability statements

Currently, the data are publicly available on Zenodo. The code is available at: https://git.wur.nl/standards/aspergillus-data-standardisation. The code used to transform the datasets, and the configuration of the FAIR-DS can be found under https://git.wur.nl/standards. The FAIR-DS formatted datasets can be found on Zenodo: the Kortenbosch et al., dataset: https://doi.org/10.5281/zenodo.14021467, and the Santos-Silva et al., dataset: https://doi.org/10.5281/zenodo.14021624. More information about the application of the FAIR- DS in the mycology field can be found here https://www.aspar-kr.site/.
