# Supplementary material for: An approach to making life sciences FAIR—FAIR-DS as a tool for Aspergillus fumigatus
Source: Database (Oxford). 2026 Jan 2;2025:baaf082. doi: 10.1093/database/baaf082 (PMC12757778; doi:10.1093/database/baaf082)
Supplement: baaf082_Supplemental_File [file baaf082_supplemental_file.docx]

|  |  |  |  |  |  |  |
| --- | --- | --- | --- | --- | --- | --- |
| Traits of each method | ELN | EXCEL | COPO  [[13]](#_bookmark18) | CEDAR  [[14]](#_bookmark19) | ISA-tab  [[27]](#_bookmark32) | FAIR-DS  [[16]](#_bookmark21) 2.0 |
| Full workflow coverage | Partial | No | Yes | Partial | Partial | Yes |
| Enables cross-domain collaborations | Partial | Partial | Partial | Partial | Partial | Yes |
| Possibility to edit offline | Yes | Yes | No | No | Partial | Yes |
| Long-term archiving | Yes | No | Yes | No | Partial | Yes |
| FAIR (meta)data standardized formats | No | No | Partial | Yes | Yes | Yes |
| Protocols/guidelines available online | Yes | Yes | Partial | Partial | No | Yes |
| Operable as a standalone system | Yes | Yes | Partial | Partial | Yes | Yes |
| Data exportable in interoperable format | Yes | Yes | Partial | Partial | No | Yes |
